# Forced sexual intercourse and its association with HIV status among people attending HIV Voluntary Counseling and Testing in a healthcare center in Kinshasa (DRC)

**DOI:** 10.1371/journal.pone.0189632

**Published:** 2017-12-18

**Authors:** Eduardo Burgueño, Silvia Carlos, Cristina Lopez-Del Burgo, Alfonso Osorio, Maria Stozek, Adolphe Ndarabu, Philémon Muamba, Philomene Tshisuaka, Jokin De Irala

**Affiliations:** 1 CEFA-Monkole, Kinshasa, Democratic Republic of the Congo; 2 School of Medicine, University of Mwene-Ditu, Mwene-Ditu, Democratic Republic of the Congo; 3 Preventive Medicine and Public Health Department, University of Navarra, Pamplona, Spain; 4 IdiSNA, Navarra Institute for Health Research, Pamplona, Spain; 5 Institute for Culture and Society (ICS), University of Navarra, Pamplona, Spain; 6 School of Education and Psychology, University of Navarra, Pamplona, Spain; 7 Monkole Hospital, Kinshasa, Democratic Republic of the Congo; 8 School of Law and Politics, Catholic University of Congo, Kinshasa, Democratic Republic of the Congo; 9 ISSI-Monkole Nursing School, Kinshasa, Democratic Republic of the Congo; University of Westminster, UNITED KINGDOM

## Abstract

**Introduction:**

Sexual violence, an HIV determinant, is an integrated behavior in the D.R.Congo. We aimed to analyze the prevalence of forced sexual intercourse (FSI) among people receiving HIV Voluntary Counseling and Testing in a hospital in Kinshasa, and its association with socio-demographics, behaviors and HIV status.

**Methodology:**

Case-control study (2010–2012). Two-hundred and seventy-four cases with a new HIV+ test and 1,340 controls with an HIV- test were interviewed about HIV-related knowledge, attitudes and behaviors, including FSI.

**Results:**

Thirty-four percent of the participants declared having had FSI (38% of women and 32% of men). Being a woman, aged 25–49 and reporting multiple sexual partners were associated with reporting FSI. For men, being single was protective against FSI; and cohabiting, having a high socioeconomic status, and alcohol consumption increased the odds. For women, being single, divorced/separated and widow was associated with reporting FSI. A significant positive association was found between FSI and an HIV positive test.

**Conclusion:**

Among our Congolese population, FSI was strongly associated with HIV infection and it was also associated with alcohol consumption and multiple sexual partnerships, other key HIV determinants. These behaviors need to be identified as potential risk factors of FSI during counseling interventions. Researchers, practitioners and decision-makers should work together to get violence prevention integrated into health, social and educational policies.

## Introduction

The differences between HIV epidemics around the world show the importance of analyzing the specific behavioral as well as the social and structural determinants.

In the Sub-Saharan African (SSA) region, sexual risk behaviors such as multiple sexual partnerships, transactional sex or sexual violence are at the base of the HIV infection and other sexually transmitted infections (STIs) [1–4]. At the same time, they are strongly associated with a low literacy level, poverty, corruption, and gender issues, among others [5,6].

A strong relationship between sexual violence and HIV incidence has been described [7,8] and different mechanisms have been proposed to explain this association: direct infection from FSI (anogenital trauma), limited control over sexual intercourse (i.e. condom use), multiple sexual partners as a result of separation derived from intimate partner violence (IPV), or violent partners more likely to exhibit HIV risk behaviors (i.e. commercial sex, frequent alcohol consumption, multiple sexual partners, unprotected sex or presence of STIs) [1,7,9,10,11,12–14].

In the Democratic Republic of Congo (DRC) HIV prevalence was 0.8% in 2015 among the general adult population (higher among the young population) and 1.6% in Kinshasa, the capital city, where around 10 million inhabitants live [15]. It is not among the highest HIV prevalent countries in SSA, but still millions of people are suffering the HIV/AIDS consequences. As for most African countries, heterosexual transmission is the main transmission route, mainly as a result of early sexual debut, multiple sexual partners, low frequency of consistent condom use, of transactional sex and sexual violence [16,17].

The understanding of the HIV epidemic in the DRC needs to take into account a larger vision including the socio-ecological determinants responsible of these behaviors. For example, transactional sex is an expression of the survival conditions for many people in the country, such as homelessness and financial difficulties to obtain food, clothes, healthcare or to pay student fees [10,18–21]. All of these factors are connected with the fact that the war has paralyzed the development of public policies, including public health. More international and governmental intervention is required to deal with the consequences of the war and improve the living conditions of people on DRC.

With regards to sexual violence, the DRC is one of the world regions with the highest prevalence of sexual violence, and community and family violence in general are established behaviors in the country [16, 22,23]. In the DRC 82% of children <15 years old have suffered some kind of violence, 52% of women at some moment of their life (with 13% of physical violence during the pregnancy), and up to 75% find a justification for a man to hurt his female partner [16–18,24]. Additionally, the DRC has the serious and specific problem of rapes in a context of chronic war in the East of the country [25–29]. Although the 2014 national Demographic and Health Survey analyzed the prevalence of sexual violence among women 15–49 years old (27% national and 13% in Kinshasa, the capital city), (as well as physical (52%) and psychological violence (37%) [16,24,26], there is no specific information about sexual violence among men, the healthcare and HIV testing context, the socio-structural determinants or about its association with different sexual risk behaviors or HIV infection. Therefore, we aimed to evaluate, in a population who was voluntarily tested for HIV in a district hospital in Kinshasa, the prevalence of forced sexual intercourse (FSI) and its association with socio-demographic and behavioral factors as well as with HIV infection.

## Methodology

### Study design

As previously described [30], we carried out a case-control study with a prospective recruitment of incident cases in which we analyzed data about knowledge, attitudes and behaviors related to HIV infection.

### Setting and study population

From December 2010 until June 2012, people aged 15–49 attending HIV Voluntary Counseling and Testing (VCT) at a reference hospital in Kinshasa and blood donors at the same center to whom VCT was offered, were offered to participate in the study by the VCT personnel. They had never been HIV tested before or had a previous HIV-negative result. Cases were participants with a first-time positive test for HIV. Controls were participants from the same base population that got an HIV negative test at the VCT at the study time.

### Study sample

When the study was designed, around 1,000 people aged 15–49 annually attended the study VCT center for VCT or blood donation and around 15% were HIV+ newly diagnosed. With an estimated 90% response rate, we calculated that during 18 months we could recruit about 1,350 participants and 200 new HIV+ diagnoses [31]. Considering that in logistic regressions about 10 events are needed for each parameter in the model [32], we were confident to be able to simultaneously adjust for around 20 parameters.

In order to be able to detect the differences between cases and controls in the prevalence of sexual risk behaviours (i.e. a 25% and 10% prevalence of multiple sexual partners among HIV+ cases and HIV negative controls, respectively) [33,34], we considered a 1:4 ratio of cases and controls, taking into account that during the study period the number of cases was not predicted to be high [31].

### Data collection

Participants replied to a face-to-face interview on HIV-related knowledge, attitudes and behaviors, and provided information about whether they had ever suffered or not from FSI. Interviewers were both male and female nurses trained for HIV VCT. Due to the nature of some sensitive and intimate questions, interviewers were trained for a complete comprehension of the meaning for each of the questions.

### Laboratory analysis

Participants were tested for HIV during the VCT process: pre-test counseling, HIV test and post-test counseling. According to the national protocol in Congo-Kinshasa for HIV diagnosis, rapid diagnostic routine tests were used. The first test for HIV-antibody detection was Determine^®^ HIV-1/2 (immunochromatographic test for the detection of antibodies to HIV types 1 and 2). If this was positive the blood sample was analyzed by two other rapid antibody detection tests: DoubleCheckGold^®^ and Unigold^®^ (rapid immunoassays for the qualitative detection of antibodies to HIV-1 and/or HIV-2). The laboratory analyses were carried out locally in Kinshasa. The local context did not allow to confirm ‘undetermined’ HIV tests, therefore participants with this result were excluded from the study but they were integrated for their appropriate follow-up.

### Statistical analysis

For this study we just included participants that reported having ever had sex.

FSI was evaluated with the question `Have you ever had sex against your will?´. The different answer options to this question were: ‘yes’, ‘no´, ‘I don´t know´. We recategorized this variable into `yes’ and ‘no/I don´t know’.

We first carried out bivariate analyses to evaluate the differences between cases and controls and to describe the prevalence of FSI among the study participants, considering their socio-demographical characteristics (sex, age, education, socioeconomic level and marital status), their weekly alcohol consumption (`never´, `once a week´, `once daily´, `twice daily´ and `more than twice a day´, recoded as `yes´or `no´) and sexual behaviors. With regards to the sexual behaviors that could be associated with reporting FSI, we evaluated the prevalence of multiple sexual partnerships at the study time, as well as partner´s multiple lifetime sexual partnerships. The study questionnaire included different questions for concurrent sexual partners (‘Do you actually have multiple sexual partners’ (no/yes), ‘how many partners do you have?’). The question for partner´s multiple sexual partners had four categories (`one´, `between two and five´, `more than five´ and `I don´t know´). For both variables the number of sexual partners was categorised as a dichotomous categorical variable (yes/no).

After the initial descriptive analysis, we carried out non-conditional logistic regressions to evaluate the association between FSI and an incident HIV positive result. This analysis was adjusted for the study subgroup (VCTattendees/blood donors), sociodemographics, alcohol consumption and sexual behavioral variables (multiple sexual partners, multiple partner´s partners and condom use).

An additional multivariate analysis was carried out to study the association between participants´ socio-demographics and risk behaviors with FSI. All sociodemographics characteristics, the study subgroup, alcohol consumption and sexual behavioural variables (multiple sexual partners, multiple partner´s partners). were included in the model.

All multivariate analyses were stratified by sex.

A goodness-of-fit,test was carried out in all regressions, with non-significant p values showing a good concordance between the observed and expected values.

All statistical analyses were carried out with Stata software (version 12.1). P-values <0.05 were considered significant.

### Ethical considerations

Ethical approval from the Research Ethics Committees of the two study centers were obtained. An oral informed consent was obtained from each participant consistent with local clinical practices at that point [35,36]. The oral consent was registered by one of the interviewers under the supervision of another staff member from the local HIV center–nurse, laboratory technician or doctor.

## Results

We recruited 1,630 participants (31.6% women and 68.4% men), 274 HIV positive cases and 1,340 HIV negative controls. Sixteen participants that got an undetermined HIV test and 59 participants, who had never had sex, were excluded from the analyses.

Table 1 shows the characteristics and behaviors of cases and controls and their association with HIV positivity, considering both the whole study population as well as the sample stratified of by sex. After adjusting for confounding factors, we found that participants that went to the hospital for receiving VCT (client-initiated VCT) were significantly more likely to get an HIV positive test than the blood donors that were offered to get VCT (provider-initiated VCT) (OR = 5.68; 95%CI:3.74–8.65). This effect was much stronger for women than for men. We also observed that women had a higher probability of getting and HIV positive test (OR = 1.47; 95%CI:1.00–2.17). For women, an older age (25–49 years *vs*. 15–19 years) and a widow marital status (compared with married) were significantly associated with HIV (OR = 3.37; 95%CI:1.02–11.11, and OR = 5.00; 95%CI:1.31–19.16, respectively). On the contrary, for female, having a higher education level and being single were protective against HIV infection (OR = 0.09; 95%CI:0.02–0.45 and OR = 0.44; 95%CI:0.22–0.86, respectively). With regards to the participants´ sexual behaviours, reporting having ever had FSI was associated with HIV positivity, both for women and men (OR = 2.93; 95%CI:1.67–5.17 and OR = 2.51; 95%CI:1.60–3.94, respectively). For men, reporting concurrent multiple sexual partners increased the probability of being seropositive (OR = 3.53; 95%CI:2.12–5.90). Finally, although very few participants reported always using a condom, we observed that reporting using condoms sometimes or almost always was associated with a lower probability of being HIV positive, compared to never use (OR = 0.56; 95%CI:0.40–0.78).

**Table 1:** Characteristics and behaviours of cases and controls and their association with HIV-positivity.

	Cases (N = 274) (%)	Controls (N = 1,340) (%)	HIV OR (95%CI)^a^	HIV OR (95%CI)^a^ Women	HIV OR (95%CI)^a^ Men
**Study group**					
Blood donors (n = 860)	18.2 (13.8–23.3)	60.4 (57.8–63.1)	1 (Ref.)	1 (Ref.)	1 (Ref.)
VCT (n = 754)	81.7 (76.7–86.1)	39.5 (36.9–42.2)	5.68 (3.74–8.65)	15.09 (4.83–47.13)	4.59 (2.87–7.34)
**Sociodemographics**					
Sex					
Men (n = 1,103)	47.1 (41.0–53.2)	72.7 (70.2–75.1)	1 (Ref.)	—	—
Women (n = 511)	52.9 (46.8–58.9)	27.3 (24.9–29.8)	1.47 (1.00–2.17)		
Age (years)					
15–19 (n = 145)	4.0 (2.0–7.1)	10.0 (8.4–11.7)	1 (Ref.)	1 (Ref.)	1 (Ref.)
20–24 (n = 377)	9.5 (6.3–13.6)	26.2 (23.9–28.6)	1.25 (0.51–3.05)	1.21 (0.32–4.52)	1.24 (0.34–4.51)
25–49 (n = 1,092)	86.5 (81.9–90.3)	63.8 (61.2–66.4)	2.71 (1.20–6.12)	3.37 (1.02–11.11)	1.95 (0.60–6.35)
Education					
No studies/illiterate (n = 71)	12.0 (8.4–16.5)	2.8 (2.0–3.9)	1 (Ref.)	1 (Ref.)	1 (Ref.)
Primary (n = 57)	9.1 (6.0–13.2)	2.4 (1.6–3.3)	1.20 (0.48–3.04)	0.63 (0.11–3.43)	2.36 (0.65–8.65)
Secondary (n = 1,228)	67.5 (61.6–73.0)	77.8 (75.5–80.0)	0.34 (0.17–0.66)	0.16 (0.05–0.51)	0.68 (0.24–1.90)
University (n = 258)	11.3 (7.8–15.7)	16.9 (15.0–19.1)	0.29 (0.13–0.65)	0.09 (0.02–0.45)	0.72 (0.22–2.27)
Socioeconomic level					
Low (n = 565)	47.1 (41.0–53.2)	32.5 (30.0–35.1)	1 (Ref.)	1 (Ref.)	1 (Ref.)
Middle (n = 929)	48.5 (42.5–54.6)	59.4 (56.7–62.0)	0.68 (0.47–0.97)	0.55 (0.31–0.97)	0.75 (0.47–1.22)
High (n = 120)	4.4 (2.3–7.5)	8.1 (6.7–9.6)	0.34 (0.16–0.75)	0.38 (0.11–1.33)	0.28 (0.10–0.80)
Marital status					
Married (n = 420)	33.6 (28.0–39.5)	24.5 (22.2–26.9)	1 (Ref.)	1 (Ref.)	1 (Ref.)
Single (n = 1,045)	41.6 (35.7–47.7)	69.5 (66.9–71.9)	0.57 (0.38–0.85)	0.44 (0.22–0.86)	0.65 (0.39–1.09)
Cohabitation (n = 61)	3.3 (1.5–6.1)	3.9 (2.9–5.1)	0.81 (0.35–1.88)	0.76 (0.04–15.07)	0.83 (0.34–2.04)
Divorced/separated (n = 50)	10.2 (6.9–14.4)	1.6 (1.0–2.5)	1.73 (0.83–3.60)	1.46 (0.57–3.79)	1.19 (0.27–5.15)
Widow (n = 38)	11.3 (7.8–15.7)	0.5 (0.2–1.1)	4.69 (1.82–12.08)	5.00 (1.31–19.16)	1.77 (0.27–11.46)
**Substance use**					
Alcohol consumption (weekly)					
No (n = 890)	55.5 (49.4–61.4)	55.1 (52.4–57.8)	1 (Ref.)	1 (Ref.)	1 (Ref.)
Yes (n = 724)	44.5 (38.5–50.6)	44.9 (42.2–47.6)	1.11 (0.78–1.57)	1.37 (0.77–2.44)	1.01 (0.64–1.60)
**Sexual behaviours**					
Forced sexual intercourse					
No (n = 1,082)	42.0 (36.1–48.1)	72.2 (69.7–74.5)	1 (Ref.)	1 (Ref.)	1 (Ref.)
Yes (n = 532)	58.0 (51.9–63.9)	27.8 (25.4–30.3)	2.61 (1.86–3.67)	2.93 (1.67–5.17)	2.51 (1.60–3.94)
Multiple current sexual partners					
No (n = 1,452)	79.6 (74.3–84.2)	92.1 (90.5–93.5)	1 (Ref.)	1 (Ref.)	1 (Ref.)
Yes (n = 162)	20.4 (15.8–25.7)	7.9 (6.5–9.5)	3.12 (1.97–4.95)	1.75 (0.63–4.84)	3.53 (2.12–5.90)
Partner with multiple lifetime					
partners					
No (n = 1,347)	78.8 (73.5–83.5)	84.4 (82.3–86.3)	1 (Ref.)	1 (Ref.)	1 (Ref.)
Yes (n = 267)	21.2 (16.5–26.5)	15.6 (13.7–17.6)	1.31 (0.87–1.98)	1.58 (0.86–2.93)	1.06 (0.57–1.97)
Condom use					
Never (n = 655)	61.4 (55.3–67.2)	36.7 (34.1–39.3)	1 (Ref.)	1 (Ref.)	1 (Ref.)
Sometimes (n = 859)	37.1 (31.4–43.2)	57.0 (54.3–59.7)	0.56 (0.40–0.78)	0.51 (0.29–0.90)	0.58 (0.37–0.91)
Always (n = 88)	1.5 (0.4–3.7)	6.3 (5.1–7.8)	0.34 (0.11–1.02)	NA	0.41 (0.13–1.28)

^a^ OR: Odds Ratio, CI: confidence interval. Logistic regression adjusted for all the variables in the table. Goodness-of-fit test, p = 0.1016.

NA Not applicable.

Overall, five hundred and thirty participants (34%) declared having ever had FSI: 37.8% among women and 32.4% among men. When we analyzed the differences in the prevalence of FSI among HIV+ cases and HIV- controls, we found a prevalence of 58% and 27.8%, respectively (Chi2 p<0.001).

Table 2 shows the characteristics and behaviors of the study participants associated with having ever had FSI. Among participants reporting FSI there was a significant higher odds of being a woman (OR = 1.37; 95%CI: 1.03–1.82) and being older (OR = 2.16; 95%CI:1.21–3.86 and OR = 3.31; 95%CI:1.90–5.77 for 20–24 years old and 25–49 years old compared to aged 15–19). Although no significant, for both women and men, having a higher education seemed to protect from having had FSI. Among women, compared to being married, being single, divorced/separated and widow was significantly associated with a higher odds of reporting FSI (OR = 2.57; 95%CI:1.43–4.60, OR = 3.58; 95%CI:1.56–8.22 and OR = 2.82; 95%CI:1.16–6.88, respectively). For men, being single was protective against FSI (OR = 0.56; 95%CI:0.40–0.78) and cohabitation was on the contrary, associated with a higher probability of reporting FSI (OR = 2.57; 95%CI:1.39–4.76), For males, reporting a higher socioeconomic level was associated with FSI (OR = 2.23; 95%CI:1.24–4.04). In this group, alcohol consumption was associated with FSI (OR = 1.84; 95%CI: 1.38–2.45). With regards to sexual risk behaviors, for both men and women, having multiple concurrent sexual partners was significantly associated with reporting unwanted sex (OR = 3.16; 95%CI:2.20–4.53).

**Table 2:** Prevalence of forced sexual intercourse (FSI) among study participants and characteristics and behaviours associated with FSI.

	N	FSI (N = 530) % (95%CI)	FSI OR (95%CI)^a^	FSI OR (95%CI)^a^ Women	FSI OR (95%CI)^a^ Men
**Study group**					
Blood donors	823	28.7 (25.6–31.8)	1 (Ref.)	1 (Ref.)	1 (Ref.)
VCT	732	40.2 (36.6–43.7)	1.55 (1.19–2.02)	1.25 (0.72–2.17)	1.59 (1.16–2.18)
**Sociodemographics**					
Sex					
Men	1068	32.4 (29.6–35.2)	1 (Ref.)	—	—
Women	487	37.8 (33.5–42.1)	1.37 (1.03–1.82)		
Age (years)					
15–19	121	14.9 (8.4–21.3)	1 (Ref.)	1 (Ref.)	1 (Ref.)
20–24	357	26.0 (21.5–30.6)	2.16 (1.21–3.86)	2.86 (1.27–6.46)	2.01 (0.86–4.69)
25–49	1077	38.9 (36.0–41.8)	3.31 (1.90–5.77)	4.13 (1.90–8.96)	3.12 (1.38–7.08)
Education					
No studies/illiterate	68	38.2 (26.4–50.1)	1 (Ref.)	1 (Ref.)	1 (Ref.)
Primary	56	58.9 (45.6–72.2)	2.42 (1.13–5.20)	5.94 (1.58–22.31)	1.79 (0.64–4.99)
Secondary	1181	32.3 (29.7–35.0)	0.91 (0.52–1.57)	0.94 (0.42–2.12)	0.91 (0.41–2.00)
University	250	35.6 (29.6–41.6)	0.82 (0.43–1.53)	0.65 (0.24–1.80)	0.84 (0.35–1.99)
Socioeconomic level					
Low	545	32.5 (28.5–36.4)	1 (Ref.)	1 (Ref.)	1 (Ref.)
Middle	894	33.5 (30.5–36.7)	1.19 (0.92–1.54)	0.77 (0.49–1.21)	1.41 (1.02–1.94)
High	116	45.7 (36.5–54.9)	2.16 (1.35–3.48)	2.10 (0.91–4.85)	2.23 (1.24–4.04)
Marital status					
Married	413	38.7 (34.0–43.5)	1 (Ref.)	1 (Ref.)	1 (Ref.)
Single	993	29.0 (26.2–31.8)	0.82 (0.61–1.08)	2.57 (1.43–4.60)	0.56 (0.40–0.78)
Cohabitation	61	55.7 (42.9–68.6)	2.48 (1.40–4.40)	—	2.57 (1.39–4.76)
Divorced/separated	50	56.0 (41.7–70.2)	1.85 (0.98–3.46)	3.58 (1.56–8.22)	1.72 (0.54–5.51)
Widow	38	52.6 (36.0–69.3)	1.39 (0.68–2.85)	2.82 (1.16–6.88)	0.78 (0.13–4.53)
**Substance use**					
Alcohol consumption (weekly)					
No	846	27.9 (24.9–30.9)	1 (Ref.)	1 (Ref.)	1 (Ref.)
Yes	709	41.5 (37.8–45.1)	1.73 (1.37–2.18)	1.51 (0.98–2.32)	1.84 (1.38–2.45)
**Sexual behaviours**					
Multiple current sexual partners					
No	1393	31.2 (28.8–33.7)	1 (Ref.)	1 (Ref.)	1 (Ref.)
Yes	162	58.6 (51.0–66.3)	3.16 (2.20–4.53)	4.03 (1.60–10.13)	2.81 (1.88–4.20)
Partner with multiple lifetime					
partners					
No	1288	34.5 (31.9–37.1)	1 (Ref.)	1 (Ref.)	1 (Ref.)
Yes	267	32.2 (26.6–37.8)	0.90 (0.66–1.22)	1.36 (0.86–2.16)	0.70 (0.46–1.08)

^a^ OR: Odds Ratio, CI: confidence interval. Logistic regression adjusted for all the variables in the table. Goodness-of-fit,p = 0.0848.

## Discussion

The most reported forms of violence in SSA countries are physical and psychological violence. Sexual violence is the least reported [3,6,25]. Among our study participants, 34% reported having ever had FSI (32% and 38% among men and women, respectively). Although our prevalence corresponds to the figures estimated for most SSA countries [10, 37,38] it is higher than the figures described for women in Kinshasa in the 2014 national Demographic and Health Survey (DHS), in which 16% of women reported having suffered sexual violence (the same frequencies described in the previous 2007 DHS) [16,33]. Many of our participants were attending a healthcare center for HIV VCT and therefore, it was a higher risk population, probably more likely to report risk behaviors than the general population.

As described in many other low-income countries [10,38–40], the prevalence of FSI in our study was significantly higher among women. African cultural practices and belief systems provide the social environment for men to consider women as a property [41]. At the same time, women are socialized to believe that men are superior to them, and also accept violence as a way of discipline [6,42–46]. Cultural theorists point to the determinants that lead to unequal power relations (differential socialization patterns, forced marriages) as some of the processes that make some African men abuse their partners. With regards to sexual violence among men, although in SSA men are usually considered as perpetrators [47], they can also be victims [48]. There are few data on the prevalence of men’s experience of sexual violence but national estimates between 6% and 27% have been described for different Southern African countries [1,49–52]. There are almost no data available from Kinshasa regarding the prevalence of sexual violence suffered by men. The national DHS mostly shows data on women, except for some information on the prevalence of women being physically violent against their partners (7.4% and 9.4% in Kinshasa) [16]. In another study carried out in the DRC a prevalence of 4% of male victims was described [53]. In our study we found that 32% of men reported having suffered from FSI. Therefore, both men and women need to be involved in activities to work on violence and the underlying gender norms [54].

In our study, older adults (25–49 years old) were significantly more likely than younger participants to have reported having had sex against their will. We did not ask about the age at which they had coerced sex. A recent paper on IPV among South African pregnant HIV+ women showed that although emotional and physical violence were more frequent among 18–24 years old, sexual violence was more prevalent among 25–29 years old women [55]. A comparative study including DHS data from 13 SSA countries found in all countries that women‘s age was significantly and strongly associated with their acceptance of wife beating [56]. Maybe it is not just the age alone, but also other age-related factors, such as the age difference between the couple members [51,56,57], which could have a stronger impact on sexual violence suffering or reporting.

An unexpected finding was the association between reporting FSI and perceiving a high personal economic status (this was only significant for men). Lower economic levels may increase the risk of violence as a result of women´s economic dependence on men, or violence perpetuation as a consequence of men´s low income or low job status [6,58]. However, some studies have also shown an association between a higher economic status and violence [6,29].

We did not find a statistically significant association between level of education and FSI, although a protective effect seems to be apparent for higher levels. Although a higher education can have an impact on decision making [56] and consequently on the capacity of refusing forced sex, apart from education empowerment can protect against sexual violence [3]. The partner´s education level can also influence this association [6,59,60].

With regards to the marital status of the participants, consistent with other studies [61–63], we found that, for men, those reporting cohabitation were significantly more likely to report FSI compared to married participants. Some possible explanations have been suggested, such as: the lower or absent legal protection among cohabitors; the lower socioeconomic status of the couple [64]; the weak social cohesion as a result of a more temporal and unstable character of the cohabiting status; the lower security of their relationship [61,63]; or the different objectives, lifestyles and personal characteristics of the people who choose cohabitation [63,65]. Therefore, the cohabitation status needs to be considered, especially now that the prevalence of cohabitation in the DRC has increased (15% in 2014, compared to 7% in 2007) [16,33]. For women being single, separated/divorced and widow, compared to being married, increased their vulnerability of having suffered FSI, as shown in the 2016 South-African DHS [66].

We also found an independent higher risk of FSI among men reporting consumption of alcohol at least once a week. According to the 2014 DHS from the DRC, among women reporting sexual violence, 31% said that their partner drank `often´. It has been widely shown that alcohol is a risk factor for sexual violence and viceversa [10,11,37,38,67]. Alcohol may cause aggressive behaviors as a result of a distortion in the perceptions or of a disinhibition [6], it can also make it more difficult for the victims to protect themselves or it can even bring the victims to places where they have a greater risk of meeting a potential offender [3,9,68–70]. It is a relevant aspect to be considered in sexual violence prevention and in HIV VCT for adults and young people in the DRC, where a high percentage of adolescents both use alcohol and are sexually active [9].

As described by other authors [3,67,71–76], reporting multiple sexual partnerships was associated with FSI. This association has been described both for suffering and perpetrating sexual violence. Perpetrators in casual relationships have been described to have had more sexual partners, to be more dominant, to be less likely to have planned sex, and to disagree with the woman’s interest for longer relationships. Additionally, men having casual sex are more likely to pressure previous partners they feel belong to them [45].

Finally, we found a strong association between reporting FSI and HIV infection, as shown in many other studies, which also have described an association with other sexually transmitted infections [4,10,38,77,78]. Different mechanisms have been suggested: vaginal and anal injuries as a result of forced sex, immune system deregulation and dysfunction, increased vaginal washing and drying or increase in sexual risk behaviors as a consequence of coerced sex [77,79–83]. The relation between violence in general and HIV and other STIs is bidirectional [3,75], as being infected can also lead to violence, as a result of disclosure and stigma-related problems [82]. Therefore, preventive programs need a coordinated and multi-sectorial approach, which includes both violence and HIV-related contents [84].

Health care settings can be a good option to integrate sexual violence and HIV prevention services and HIV counseling can be a good opportunity for empowerment and support [13,54,67]. Additionally, antiretroviral providing centers can help with sexual violence prevention and control, as it has been reported that violence is also associated with a lower attendance [8] and lower treatment adherence among HIV positive participants [85]. Apart from the healthcare-centers, the engagement of the Congolese government, NGOs, social movements, and others actors is urgently needed in order to reduce the prevalence of the structural and interpersonal factors associated with FSI. Researchers, practitioners and decision-makers should work together to get violence prevention integrated into health, social and educational policies [3].

The present study has some limitations. First, all the information collected was based on self-report which may lead to unreported events due to stigmatization [25]. However, we observed similar prevalences of FSI in Kinshasa than in other African studies and even if under-reporting was present, we found prevalences high enough to highlight the need of urgent prevention of sexual violence. In any case, the questionnaire was anonymous, which likely would result in more reporting of FSI. Also the interviews took place in a private place and the interviewers had received specific training about how to deal with sensitive questions. Secondly, we asked about the presence of FSI in general, without specifying whether participants had been exposed to physical or psychological violence or whether it had been related with a sexual partner. Finally, we did not ask about the specific time at which FSI had taken place but taking into account local research, forced sex in adulthood is likely to have occurred in our study population.

Despite these limitations, this study has several strengths. First, this is the first analytical study in the DRC that has analyzed the association of FSI both with socio-demographic and behavioral characteristics, and with and the participants´ HIV serostatus. Secondly, thanks to our large sample size (1,614 Congolese participants) we could estimate relevant adjusted associations.

## Conclusion

Forced sexual intercourse (FSI) has a strong association with HIV infection. At the same time, it is associated with being a woman, being in a cohabitation relationship, being an alcohol consumer and having multiple sexual partnerships, other key HIV determinants in Kinshasa. Therefore, these associated behaviors need to be identified by qualified HIV counselors as potential risk factors of FSI during counseling interventions and to be mentioned in groups when determinants of HIV infection are discussed in prevention programs. There is an urgent need of programs and policies focused on the reduction of the prevalent FSI in this population.
